# An increased cell cycle gene network determines MEK and Akt inhibitor double resistance in triple-negative breast cancer

**DOI:** 10.1038/s41598-019-49809-3

**Published:** 2019-09-16

**Authors:** Vera E. van der Noord, Ronan P. McLaughlin, Marcel Smid, John A. Foekens, John W. M. Martens, Yinghui Zhang, Bob van de Water

**Affiliations:** 1 Division of Drug Discovery and Safety, Leiden Academic Center for Drug Research, Leiden University, PO box 9502, 2300 RA Leiden, The Netherlands; 2 Department of Medical Oncology, Erasmus MC Cancer Institute, Erasmus University Medical Center, PO Box 2040, 3000 CA Rotterdam, The Netherlands

**Keywords:** Breast cancer, Cancer therapeutic resistance

## Abstract

Triple-negative breast cancer (TNBC) is an aggressive subtype of breast cancer with poor clinical prognosis and limited targeted treatment strategies. Kinase inhibitor screening of a panel of 20 TNBC cell lines uncovered three critical TNBC subgroups: 1) sensitive to only MEK inhibitors; 2) sensitive to only Akt inhibitors; 3) resistant to both MEK/Akt inhibitors. Using genomic, transcriptomic and proteomic datasets of these TNBC cell lines we unravelled molecular features associated with the MEK and Akt drug resistance. MEK inhibitor-resistant TNBC cell lines were discriminated from Akt inhibitor-resistant lines by the presence of PIK3CA/PIK3R1/PTEN mutations, high p-Akt and low p-MEK levels, yet these features could not distinguish double-resistant cells. Gene set enrichment analyses of transcriptomic and proteomic data of the MEK and Akt inhibitor response groups revealed a set of cell cycle-related genes associated with the double-resistant phenotype; these genes were overexpressed in a subset of breast cancer patients. CDK inhibitors targeting the cell cycle programme could overcome the Akt and MEK inhibitor double-resistance. In conclusion, we uncovered molecular features and alternative treatment strategies for TNBC that are double-resistant to Akt and MEK inhibitors.

## Introduction

Triple-negative breast cancer (TNBC) is an aggressive subtype of breast cancer that is defined by the absence of the estrogen receptor (ER), progesterone receptor (PR) and the lack of overexpression or gene amplification of the human epidermal growth factor receptor 2 (HER2)^1^. TNBC comprises a clinically and molecularly heterogeneous group of breast cancers and accounts for approximately 15% of all breast cancer cases. Although TNBC patients more often show a complete response after neo-adjuvant chemotherapy and surgery compared to other breast cancer subtypes, still >60% of the patients do not achieve a complete remission^2^. Due to the aggressive nature of this disease and the lack of targeted therapy, these unresponsive TNBC patients have worse event-free and overall survival rates compared to other subtypes^3^. Developing effective targeted therapies for TNBC patients is thus crucial for reducing the mortality rate of this aggressive subtype.

TNBC tumours commonly have either activating mutations or amplifications of PIK3CA, BRAF, KRAS and/or EGFR and/or PTEN loss, causing abnormal activity of the Raf/MAPK/ERK or PI3K/Akt/mTOR pathway^4,5^. Inhibition of these pathways using MEK and/or Akt inhibitors is thus an attractive strategy for treating TNBC^6^. However, resistance to these inhibitors is often observed in pre-clinical studies^7–10^. Efforts to overcome this resistance include reversing possible drug-induced loss of negative feedback, by combining MEK and Akt or PI3K inhibitors, or one of these with epidermal growth factor receptor (EGFR) inhibitors^7–13^. These strategies have now reached clinical trials, but their benefits remain elusive^13,14^. Another challenge is the prediction of response to provide patients with optimal, personalised, treatment strategies. Various studies demonstrate that mutations in RAS and BRAF are associated with MEK inhibitor sensitivity and mutations in RAS, PIK3CA or PTEN are associated with Akt inhibitor sensitivity^7–9^. Yet, these features do not completely correlate with sensitivity in (pre-)clinical studies in TNBC and other malignancies^13^. Novel insights in predicting the response and providing alternatives to drug-resistant tumours are thus essential in facilitating the clinical development of such inhibitors in TNBC.

In this study, comparing the efficacy of various kinase inhibitors with multiple targets, we found that TNBC cell lines respond differentially to MEK and Akt inhibitors. By assessing TNBC cell line groups that had different response patterns to these inhibitors, we have shown that Akt or MEK inhibitor sensitivity can be distinguished by the presence of mutations in the PI3K pathway and activity of the PI3K and MAPK pathway. By performing a large-scale gene set enrichment analysis, we found that Akt and MEK inhibitor double resistance is associated with elevated expression of cell cycle genes. On the other hand, MEK and Akt inhibitor double-resistant cell lines were more, but not exclusively, sensitive to inhibitors of this network, such as dinaciclib and flavopiridol, which may constitute alternative treatment options for this TNBC subgroup. Our results thus shed light on a gene network associated with resistance to MEK and Akt inhibitors and provide potential markers of response as well as alternative treatments.

## Results

### TNBC cell lines cluster into three groups based on responses to MEK and Akt inhibitors

**Figure 1:**
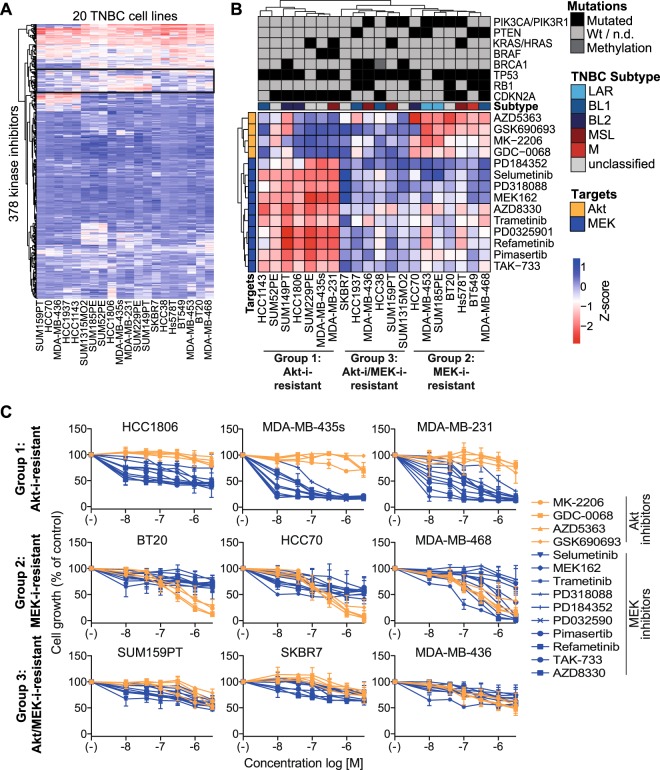
Proliferation of TNBC cell lines in response to MEK and Akt inhibitors. (**A**) A heatmap of proliferative responses of TNBC cell lines to kinase inhibitors (1 µM). Low Z-scores (red) indicate reduced cell growth, whereas high Z-scores indicate resistance (blue). The black box highlights mainly MEK and Akt inhibitors. (**B**) Clustering of responses to Akt and MEK inhibitors from this black box of TNBC cell lines among Group 1 (Akt-i-resistant), Group 2 (MEK-i-resistant) and Group 3 (Akt-i/MEK-i-resistant) cell lines. Abbreviations of TNBC subtypes: luminal androgen receptor (LAR), basal-like 1 (BL1), basal-like 2 (BL2), mesenchymal stem-like (MSL), mesenchymal (M). (**C**) Dose responses of TNBC cell line groups to the Akt and MEK inhibitors (0.01–3.16 µM). Data are expressed as means ± SD from two independent experiments.

To explore heterogeneity in targeted therapy responses, we evaluated the proliferative responses to 378 kinase inhibitors in 20 TNBC cell lines. To capture the heterogeneity in TNBC disease, these cell lines comprised multiple TNBC classes, including the basal-like, mesenchymal-like and luminal androgen receptor subtypes^15^. The TNBC cell lines largely demonstrated resistance to these inhibitors, but some inhibitors differentially affected the proliferation of the cell lines (Fig. 1A, Supplementary Dataset 1). In particular the responses to inhibitors of MEK and Akt were heterogeneous (Fig. 1A, black box, Supplementary Fig. S1), classifying the TNBC cell lines into three subgroups (Fig. 1B): cell lines were either resistant to Akt inhibitors, but sensitive to MEK inhibitors (Group 1, Akt-i-resistant); resistant to MEK inhibitors, but sensitive to Akt inhibitors (Group 2, MEK-i-resistant); or resistant to both Akt and MEK inhibitors (Group 3, Akt-i/MEK-i double-resistant). At the used concentration of 1 µM, we did not find a clear clustering of the related Raf and PI3K inhibitors (Supplementary Dataset 1 and Fig. S2). For example, while Raf inhibitors TAK-632 and RAF265 resulted in similar effects on the TNBC cell lines as the MEK inhibitors, Raf inhibitor vemurafenib induced opposite responses.

To further validate the Akt-i/MEK-i response classifications, we treated the TNBC cell lines with a dose range of the various MEK and Akt inhibitors (Fig. 1C and Supplementary Fig. S3). MEK inhibition effectively reduced the growth of Akt-i-resistant Group 1 cells at low concentrations (IC50s~10 nM, Supplementary Table S1), but not of MEK-i-resistant Group 2 or Akt-i/MEK-i-resistant Group 3 cells. Whereas Akt inhibitors affected the cell proliferation of MEK-i-resistant Group 2 cell lines (IC50s~0.1–1 µM), Akt-i-resistant Group 1 and Akt-i/MEK-i double-resistant Group 3 cell lines did not respond to these inhibitors (IC50s > 3.16 µM).

### Activity and mutations of PI3K-MAPK pathway members distinguish between Akt-i-resistant and MEK-i-resistant cell lines

**Figure 2:**
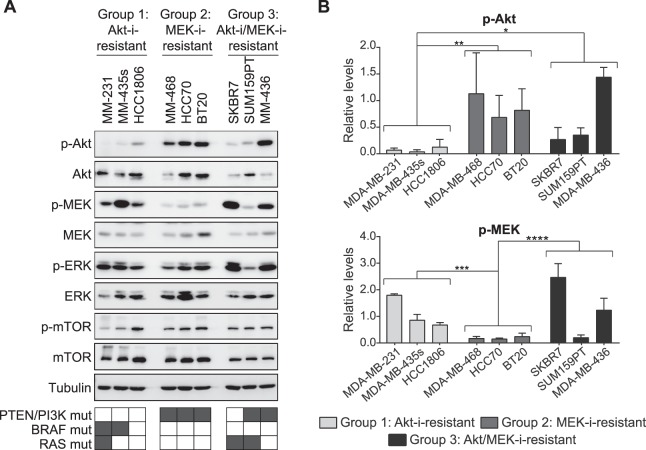
Basal levels of p-Akt and p-MEK in TNBC and drug resistance. (**A**) Western blot analysis and (**B**) quantification of p-Akt and p-MEK levels in the Akt-i-resistant, MEK-i-resistant and double-resistant cell lines. P-Akt and p-MEK levels were normalised to tubulin. Data are expressed as means ± SD of two independent experiments (two-way ANOVA, *P < 0.05, **P < 0.01, ***P < 0.001, ****P < 0.0001). Full-length uncropped blots are shown in Supplementary Fig. S4.

To understand the differential sensitivity to Akt and MEK inhibitors, we evaluated the basal phosphorylation levels of signalling components involved in the PI3K and MAPK pathways. Akt-i-resistant Group 1 cell lines expressed significantly higher levels of p-MEK compared with MEK-i-resistant Group 2 cell lines (P = 0.0003, Fig. 2A,B). In contrast, Group 2 cell lines expressed higher levels of p-Akt compared with Group 1 cell lines (P = 0.0072). Levels of p-Akt and p-MEK thus distinguish between Group 1 and 2 cell lines, suggesting that these groups exhibit a greater dependence on MAPK- or PI3K-mediated signalling, respectively, which may therefore account for the differential sensitivity of these groups to MEK and Akt inhibitors. Akt-i/MEK-i-resistant cell lines showed an average trend of increased p-Akt compared with Group 1 (P = 0.0315) and increased p-MEK compared with Group 2 (P < 0.0001) cell lines, respectively. However, Akt-i/MEK-i-resistant Group 3 cell line SUM159PT had low levels of p-MEK, indicating that this average trend is not completely discriminative. Therefore, p-Akt and p-MEK levels alone cannot discriminate between Akt-i/MEK-i double-resistant and MEK- or Akt-i-sensitive cell lines.

Next, we evaluated the mutations present in the relevant genes in the three groups to further understand the differential phenotypes. All MEK-i-resistant Group 2 cell lines with high levels of p-Akt had mutations in PTEN, PIK3CA and/or PIK3R1 (6/6) (Supplementary Table S2). These mutations were completely absent in the Akt-i-resistant Group 1 cell lines (0/7). Akt-i/MEK-i-resistant Group 3 cell lines had a mixed mutational profile (5/7). In addition, Group 1 cell lines exhibited a higher frequency of BRAF mutations upstream of MEK (2/7), whereas these were absent in both Group 2 (0/6) and Group 3 (0/7) cell lines. Mutations in KRAS or HRAS did not differentiate between any of the groups (Group 1: 2/7, Group 2: 1/6 and Group 3: 2/7). Altogether, these features could distinguish between Akt-i and MEK-i resistance, but do not explain Akt-i/MEK-i double resistance.

### Crosstalk between Akt and MEK is not essential for Akt-i/MEK-i resistance

**Figure 3:**
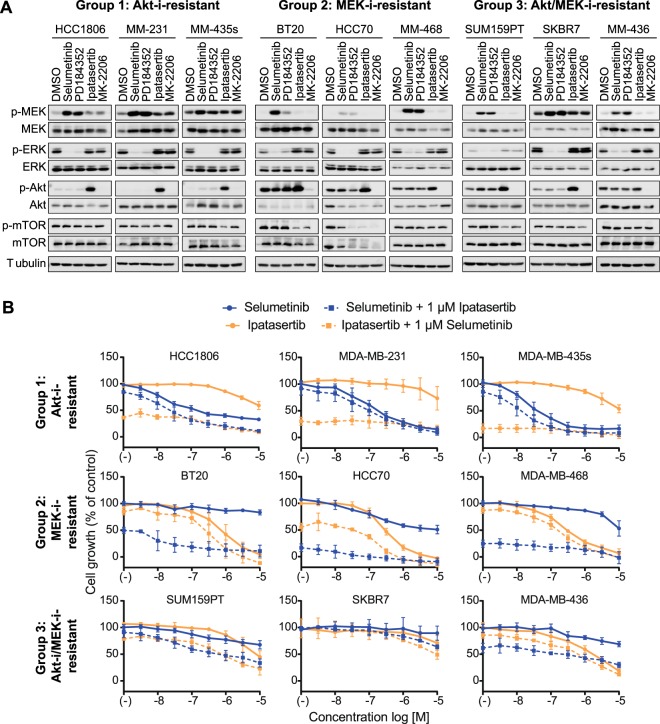
Cooperation of Akt and MEK signalling in TNBC proliferation. (**A**) Western blot analysis after inhibition by MEK inhibitors (selumetinib and PD184352) and Akt inhibitors (ipatasertib and MK-2206). Cells were treated with inhibitors (1 µM) for 24 hours. Data are representative of two independent experiments. (**B**) Combined inhibition by increasing concentrations of selumetinib or ipatasertib (0.00316–10 µM), together with a fixed concentration of ipatasertib or selumetinib (1 µM), respectively. Data are expressed as means ± SD from three independent experiments. Full-length uncropped blots, including internal controls to compare across blots, are shown in Supplementary Fig. S4.

To further explore this Akt-i/MEK-i double-resistant phenotype, we investigated the role of crosstalk between Akt and MEK pathways. If crosstalk would drive the resistant phenotype, the double-resistant, but not single-resistant, cell lines would show increased levels of active MEK/ERK upon Akt inhibition, and vice versa. Moreover, an improved, synergistic, response would be expected upon combination treatment. Treatment with ATP non-competitive MEK inhibitors selumetinib and PD184352 resulted in accumulation of p-MEK accompanied by inhibition of ERK phosphorylation in all cell lines (Fig. 3A). The extent of inhibition of ERK phosphorylation was similar across the cell line panel, indicating that MEK-i resistance is not related to a lack of target inhibition. The allosteric Akt1/2/3 inhibitor MK-2206 fully prevented Akt phosphorylation in all cell lines, whilst ATP-competitive Akt1/2/3 inhibitor ipatasertib caused accumulation of p-Akt, as binding of this inhibitor to the active site protects Ser473 phosphorylation sites from phosphatases^16^. This implies that resistance to these agents is also not due to a lack of target inhibition. The Akt inhibitors reduced p-mTOR levels in some, but not all cell lines, an event which was independent of Akt-i or MEK-i sensitivity status, suggesting mTOR activity is not critical in conferring resistance to such inhibitors. Akt inhibition slightly increased p-MEK levels in Akt-i-resistant Group 1 cell lines, but not in Akt-i/MEK-i-resistant Group 3 cell lines.

Next, we evaluated whether combining MEK inhibitors with Akt inhibitors enhances the growth-inhibitory effects of either drug. Cell lines were treated with a combination of selumetinib (MEK-i) and ipatasertib (Akt-i). The combined treatment of a dose range of selumetinib, together with a fixed concentration of ipatasertib (1 µM), and vice versa, did not overtly enhance the anti-proliferative effects of either agent in most cell lines (Fig. 3B). The combination treatments moderately sensitised SUM159PT to either selumetinib or ipatasertib. For all of the Akt-i/MEK-i-resistant Group 3 cell lines, combination treatments were incapable of reducing cell proliferation to the same extent as in the Group 1 or Group 2 cell lines. Altogether, these results suggest that crosstalk or additive ERK and Akt proliferation signalling programs are not essential for the resistance observed in Akt-i/MEK-i-resistant Group 3 cell lines.

### Akt-i/MEK-i-resistant cell lines can be distinguished by an elevated cell cycle gene expression network

**Figure 4:**
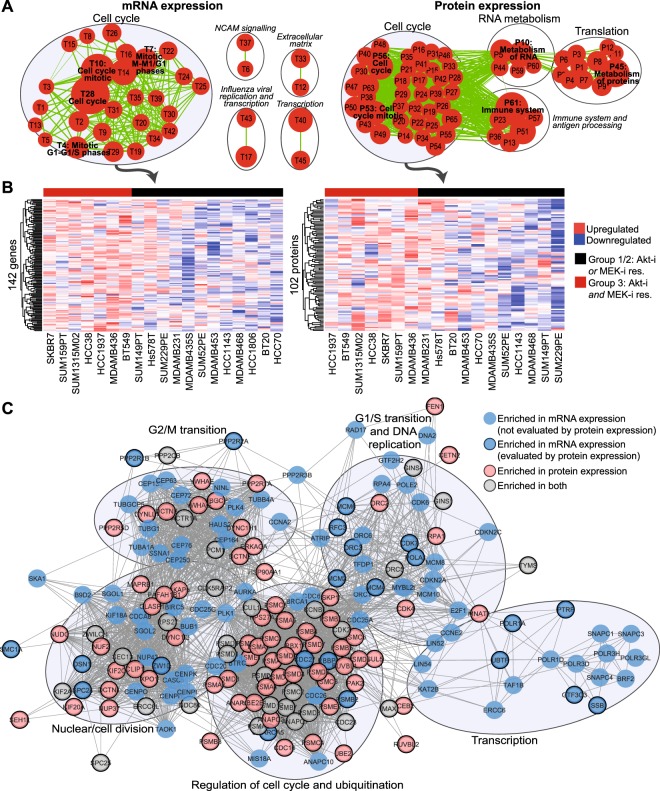
Gene set enrichment analysis (GSEA) in the Akt-i/MEK-i-resistant cell lines. (**A**) mRNA (left) and protein (right) expression were compared between MEK and Akt inhibitor-resistant Group 3 versus MEK or Akt inhibitor-resistant Group 1 and 2 cell lines using GSEA. The enrichment maps visualise enriched gene sets (P < 0.005, FDR Q-value < 0.1, overlap > 0.7), among which cell cycle gene sets were most abundant. Every node (red) represents a single overexpressed gene set and the size of the node indicates the size of the gene set. Every line (green) represents overlap between gene sets. Node numbers represent the corresponding gene set as listed in Supplementary Dataset 2. (**B**) Heatmaps of mRNA (left) and protein expression (right) of genes (log2 values and row-scaled) from the enriched genes from the cell cycle gene sets (signal-to-noise ratio >0.1). (**C**) Overlapping networks of 102 protein (pink) and 142 mRNA (blue) expression enriched cell cycle genes indicate unique and shared (grey) genes. Some genes evaluated by mRNA expression were not included in the proteomics dataset and are indicated without border.

As a next step we explored the possible signalling pathways that contribute to the double-resistant phenotype of the TNBC cell lines. Therefore, we systematically compared the previously established transcriptomes and proteomes of the three different TNBC cell panel groups. Gene set enrichment analysis (GSEA) revealed enriched gene sets in Akt-i/MEK-i-resistant Group 3 cell lines compared with both Akt-i-resistant Group 1 and MEK-i-resistant Group 2 cell lines (Fig. 4A, Supplementary Figs S5 and S6 and Dataset 2, respectively). The 37 and 62 significantly enriched gene sets (p < 0.005, FDR q-value < 0.1), based on mRNA and protein expression respectively, predominantly consisted of cell cycle-related gene sets. GSEA of protein expression data also revealed other enriched gene sets involved in translation, RNA and protein metabolism and the immune system. These gene sets overlapped (in)directly with the cell cycle related gene sets.

Within these significantly enriched cell cycle-related gene sets, we selected and explored the genes that contributed to the enrichment result (signal-to-noise ratio > 0.1) for both the transcriptomics (142 genes) and proteomics (102 genes) datasets (Fig. 4B,C, Supplementary Table S3). These genes contribute to a variety of cell cycle processes, such as G1/S transition, G2/M transition, cell cycle regulation, ubiquitination and nuclear division, suggesting that the observed cell cycle enrichment is not due to a prolonged state of one specific cell cycle phase. The genes included, amongst others, cyclin-dependent kinases (CDK) 2, 4, 5, 6 and 9, cell-division cycle (CDC) 25 A and polo-like kinases (PLK) 1, 2 and 4.

**Figure 5:**
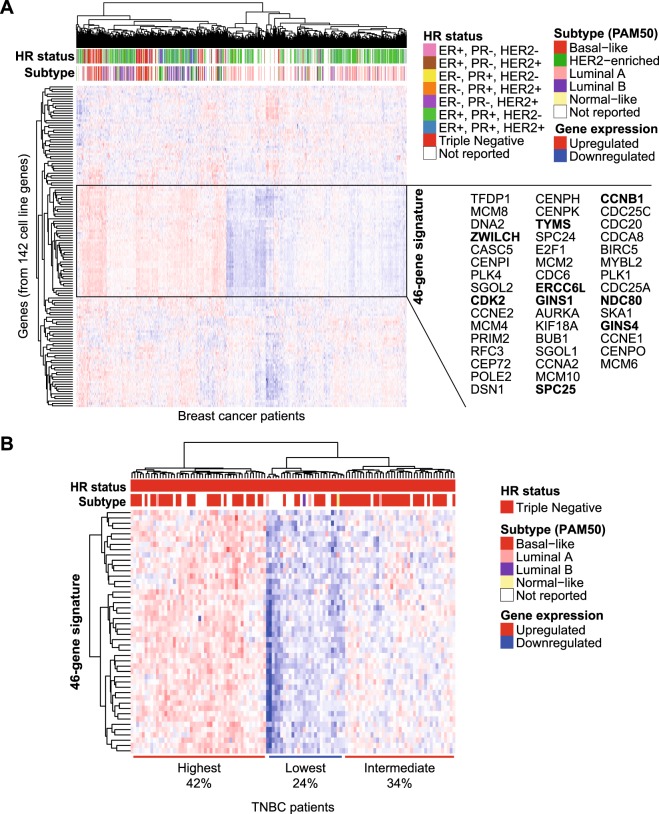
Expression of the Akt-i/MEK-i-resistant cell cycle gene network in breast cancer patients. (**A**) Clustering of all breast cancer patients (n = 1093) from the TCGA database by gene expression levels of 134 expressed genes out of the 142 mRNA cell cycle genes found overexpressed in the Akt-i/MEK-i-resistant cell lines. The black box shows a cluster of 46 genes that was consistently expressed at a higher level in one of the two subsets of patients. Genes in bold indicate overexpressed genes that were shared in mRNA and protein expression GSEA of the Akt-i/MEK-i-resistant cell lines. Colour annotations indicate hormone receptor (HR) status and PAM50 subtypes of the patients. (**B**) Clustering of TNBC patients (n = 120) from the TCGA database based on expression of these 46 genes.

Next, to examine whether the elevated mRNA expression of these 142 selected cell cycle-related genes was reflected in patient tumours, we exploited expression data from The Cancer Genome Atlas (TCGA). Hierarchical clustering uncovered a specific set of 46 genes that was consistently expressed at higher levels in a subgroup of the breast cancer patients (Fig. 5A). This cluster mainly consisted of tumours from basal-like, HER2-enriched and luminal B PAM50 subtypes. Although most of the TNBC tumours had high expression of these genes, also within the TNBC subset, these genes were differentially expressed (Fig. 5B). The hierarchical clustering revealed three clusters of TNBC tumours, with either high (42%), intermediate (34%) or low (24%) expression of the genes. The 46 genes selected from these differential cluster in patients still also subdivide TNBC cell lines in a similar manner as the original 142 genes (Supplementary Fig. S7).

### Elevated cell cycle gene expression network provides novel targets for bypassing Akt-i/MEK-i resistance

**Figure 6:**
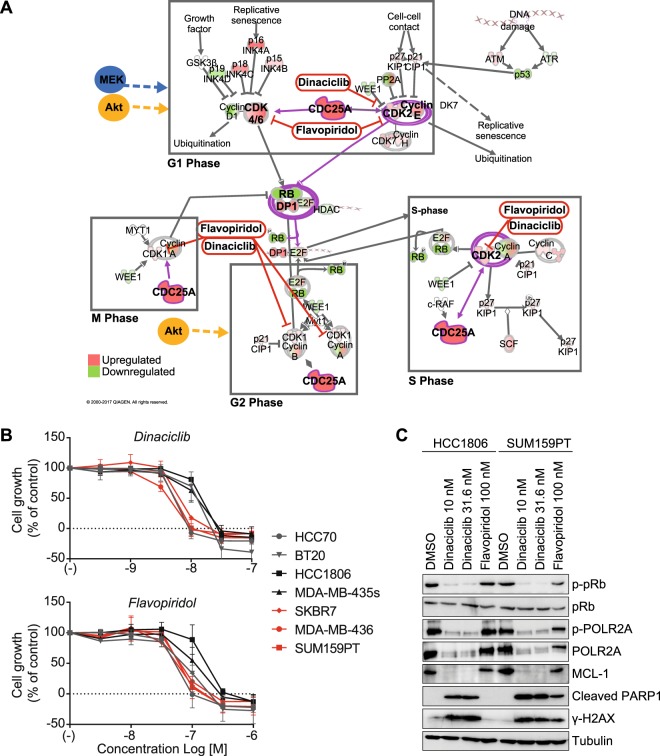
Enriched cell cycle gene expression network provides alternative targets for Akt-i and MEK-i-resistant TNBC cell lines. (**A**) Cell cycle pathway (IPA) and possible alternative drugs acting in this pathway. Green coloured targets indicate upregulation, whereas red coloured targets indicate downregulation in the Akt-i/MEK-i double-resistant cell lines. Purple circles indicate complexes with genes present in the 46-genes cluster from Fig. 5A. (**B**) Concentration response effect of the cyclin-dependent kinase (CDK) inhibitors dinaciclib and flavopiridol in Akt-i/MEK-i-resistant Group 3 cell lines (SKBR7, MDA-MB-436 and SUM159PT), Group 1 (HCC1806 and MDA-MB-435s) or Group 2 (HCC70 and BT20) TNBC cell lines. Data are expressed as mean ± SD and are representative of three independent experiments. (**C**) Western blot analysis of dinaciclib (10 or 31.6 nM) and flavopiridol (100 nM) on CDK function and apoptosis in HCC1806 and SUM159PT cells after 24 hours treatment. Full-length, uncropped, blots are shown in Supplementary Fig. S4.

Next, we investigated whether the aberrant cell cycle gene expression network found in Akt-i/MEK-i-resistant Group 3 cell lines could be an alternative target for this subgroup. Pathway analysis of the overexpressed cell cycle genes visualised core components, including CDC25A, CDK2 and CDK4 (Fig. 6A). Moreover, elevated E2F1 and DP1, together with low expression of RB1, were central in this pathway. Targeting the downstream CDKs affected by these transcription factors and CDC25A may therefore have detrimental direct and indirect effects on TNBC cell proliferation. Indeed, CDK inhibition by flavopiridol or dinaciclib strongly inhibited the growth of the Akt-i/MEK-i-resistant Group 3 cell lines (Fig. 6B). Contrastingly, Group 1 cell lines HCC1806 and MDA-MB-435s showed a relatively reduced sensitivity to dinaciclib and flavopiridol, with an average 2-3-fold increase in IC50 for both CDK inhibitors (Fig. 6B, Supplementary Table S4). Dinaciclib at 10 nM or 31.6 nM completely inhibited phosphorylation of RB1 at Ser780, an event specifically mediated by the CDK4/6-Cyclin D1 complex, in both HCC1806 and SUM159PT cells, indicative of cell cycle arrest (Fig. 6C). Importantly, flavopiridol at 100 nM moderately reduced p-RB1 levels in double-resistant Group 3 cell line SUM159PT but not in Akt-i-resistant Group 1 HCC1806 cells. Similarly, CDK9/CDK7-mediated phosphorylation of RNA Polymerase II’s C-terminal domain (POLR2A) at Ser2/5 was abolished by dinaciclib in both cell lines whereas flavopiridol exclusively reduced POLR2A phosphorylation in SUM159PT cells. Dinaciclib induced cleavage of PARP1 in both cell lines, whilst flavopiridol-induced PARP1 cleavage was solely visible in SUM159PT cells. Concomitant depletion of BH3-only family member MCL-1 and induction of H2AX phosphorylation at Ser139 in double-resistant SUM159PT cells suggested induction of apoptosis and DNA damage response signalling. These effects were absent in HCC1806 cells after exposure to flavopiridol. Notwithstanding this differential sensitivity, the Group 2 cell lines BT20 and HCC70 were equally as sensitive to the CDK inhibitors as Group 3 cell lines (Fig. 6B). These data therefore suggest that CDK inhibitors which target the aberrant cell cycle profile are especially, but not exclusively, effective in Akt-i/MEK-i-resistant Group 3 cell lines.

## Discussion

Although various potential targets characterise TNBC tumours, no effective clinical targeted therapy is available for this aggressive disease. Using a kinase inhibitor screen in a broad panel of TNBC cell lines, we mapped the responses to inhibition of various kinase targets and found that MEK and Akt inhibitors differentially affected TNBC cell proliferation. The ERK and Akt signalling programs are critical in various aspects of cancer biology, and MEK and Akt inhibitors have entered the clinic for various types of cancer^17,18^. Moreover, TNBC is often accompanied by genetic aberrations in upstream modulators of these pathways. As drug resistance to MEK and Akt inhibitors is not fully understood, this study further focussed on exploring the heterogeneity in these responses. We here demonstrate that MEK and Akt double resistance is associated with the elevated expression of a cell cycle gene network, while inhibition thereof effectively reduced cell proliferation in these MEK and Akt inhibitor double-resistant cell lines.

In this research we have focused on studying the differential responses to MEK and Akt inhibitors, as the screening indicated the most clear and consistent differences between responses to these inhibitors among the TNBC cell lines. Although Raf and PI3K inhibitors are also promising candidates under clinical development, we did not find consistent responses upon treatment with these classes of inhibitors. This might be due to off-target effects of such inhibitors or due to different mechanisms of action. Moreover, the initial screening of the kinase inhibitor library was limited to testing the inhibitors at a single concentration of 1 µM. Therefore, we do not exclude that other classes of inhibitors may also differentially affect TNBC cell proliferation at higher or lower concentrations.

Previous studies have indicated that MEK or Akt inhibitor resistance in cancer can be caused by crosstalk between the PI3K/Akt/mTOR or RAF/MEK/ERK pathways^7,8,19^. Therefore, these studies suggest that combination therapy with MEK and Akt (or PI3K) inhibitors will reverse this resistance. In this study, we did not observe induction of the parallel pathways after Akt or MEK inhibition, with the exception of SUM159PT cells. Combination therapy also did not sufficiently sensitise the Akt- and MEK inhibitor-resistant cells to either therapy. These results suggest that crosstalk is not an essential mechanism of combined Akt-i and MEK-i resistance in TNBC. Indeed, even though various studies demonstrate synergistic effects of combination therapy, these did not disrupt cell viability completely^7,8,19^. Importantly, combination therapy with MEK and Akt inhibitors has also not shown promising results in clinical trials, as beneficial effects over single treatment are hardly obtained, instead causing more severe side effects^13,14,20^. For these reasons we sought to find another strategy to overcome the resistance in MEK-i and Akt-i-resistant cells.

Our results demonstrate that Akt inhibitor-resistant cell lines can be distinguished from MEK inhibitor-resistant lines by increased levels of p-MEK and low levels of p-Akt. Additionally, mutations in PTEN, PI3KCA and/or PI3KR1 characterise MEK inhibitor-resistant but not Akt inhibitor-resistant cell lines. Similar observations were made in other cancer types by others^7–9^. However, these features do not entirely explain the observed resistance phenotypes in our panel of TNBC cell lines. Here, we further explored the underlying mechanisms responsible for double resistance to both Akt and MEK inhibitors. To this end, we took advantage of our unique transcriptome and proteome dataset of the entire TNBC cell line panel. Using large-scale gene set enrichment analysis, we revealed a 142-gene and 102-protein cell cycle-enriched network associated with Akt and MEK inhibitor double resistance. Importantly, 46 of these genes subdivides all breast cancer patients, as well as TNBC patients, into separate groups based on their gene expression. The majority of TNBC patients had high expression of these genes, which is line with that so far, most TNBC patients are not responsive to MEK and Akt inhibitor treatments^13,14,20–23^. Yet, a minority of the TNBC patients may thus be sensitive to MEK or Akt inhibitors, depending on their p-MEK/p-Akt levels. Thus, stratification of patients in clinical trials of MEK or Akt inhibitors based on the features described in this study may reveal beneficial effects that would otherwise be neglected. However, further studies are needed to translate the associations found here to patient treatment responses. Moreover, also the luminal B and HER2-enriched hormone-positive breast cancers have a higher expression of this gene signature compared to the luminal A subtype, suggesting that for hormone-positive breast cancers the PAM50 subtyping may already be predictive. Nevertheless, as the findings in this study are based on TNBC cell lines, this needs to be explored further.

The overexpression of the cell cycle genes may render signals from Akt and MEK redundant. No studies have previously linked such a profile to Akt and MEK inhibitor resistance in TNBC. However, various studies in other cancer types have recently demonstrated the role of the cell cycle in this resistance by confirming synergy between inhibitors of the PI3K/MAPK pathway and inhibitors of the cell cycle, such as CDK and PLK inhibitors^24–30^. These findings suggest that the enhanced expression and activity of cell cycle machinery components may have an important role in resistance to Akt and MEK inhibitors, not only in TNBC, but also in other cancer types.

Our data demonstrate that inhibition of CDKs that determine cell cycle progression and transcription can effectively kill TNBC cells that are otherwise resistant to Akt and MEK inhibitors. Although our initial screening did not show clear differences in responses to CDK inhibitors at 1 µM, further evaluation at lower concentrations did reveal differential responses. For example, the pan-CDK inhibitors dinaciclib and flavopiridol inhibited proliferation of SUM159T cells that were resistant to both Akt-i and MEK-i. Interestingly, some, but not all, Akt or MEK inhibitor-sensitive cell lines were more resistant to these compounds compared to MEK-i/Akt-i double-resistant cell lines. Thus, these compounds are especially, but not exclusively, effective against this group and provide possible alternative treatments for overcoming Akt and MEK inhibitor double resistance. Notably, both lethal and sub-lethal concentrations of dinaciclib inhibited RB1 and POLR2A phosphorylation in HCC1806 cells, confirming that its relative insensitivity to this agent is not related to inefficient target inhibition, unlike flavopiridol. The superior activity of dinaciclib compared to flavopiridol was also evidenced by the complete abrogation of RB1 and POLR2A phosphorylation at concentrations ten times lower than those required for flavopiridol to elicit a moderately comparable effect, which was only visible in MEK-i/Akt-i double-resistant SUM159PT cells. As dinaciclib and flavopiridol are more effective in the MEK-i/Akt-i resistant cell lines this may suggest that also for MEK-i/Akt-i resistant TNBC patients these treatments could be alternative options. This strategy could potentially be improved by more selective CDK inhibitors that are currently being developed. Nevertheless, the increased sensitivity could expand the therapeutic window to circumvent the side effects that are associated with flavopiridol and dinaciclib.

To conclude, this study describes the molecular features that can discriminate between Akt-i and MEK-i single agent-sensitive subsets of TNBC cells as well as double agent-resistant TNBC cells. For double-resistant cells we uncovered a high expression of a cell cycle gene network. Accordingly, these double-resistant cells are relatively sensitive to inhibitors of CDKs. Moreover, a number of these genes are also differentially expressed in TNBC patients. Since our study describes clinically relevant features that can subdivide TNBC cells into their Akt and MEK inhibitor response profile, these features may also facilitate the prediction of response in patients. Future mechanistic studies may reveal the origin of the aberrant cell cycle network, which could supplement this study by providing additional alternative targets and simplified biomarkers for clinical assessment. Altogether, this and future studies could therefore lead to a biomarker-based therapeutic strategy for treating TNBC patients with Akt, MEK and/or CDK inhibitors.

## Methods

### Cell culture

Twenty human TNBC cell lines were used, including BT20, BT549, HCC1143, HCC1806, HCC1937, HCC38, HCC70, Hs578T, MDA-MB-231, MDA-MB-435s, MDA-MB-436, MDA-MB-453, MDA-MB-468, SKBR7, SUM1315M02, SUM149PT, SUM159PT, SUM185PE, SUM229PE and SUM52PE. All cells were cultured in RPMI-1640 medium containing L-glutamine and 25 mM HEPES (Gibco Fisher Scientific, Landsmeer, The Netherlands) supplemented with 10% fetal bovine serum (FBS), 25 U/mL penicillin and 25 µg/mL streptomycin (Fisher Scientific) in a humidified incubator with 5% CO2 at 37 °C.

### Cell line characteristics and annotation

To reflect the heterogeneity in TNBC, the cell lines used in this study comprised multiple TNBC classes, including the basal-like type 1 (BL1; HCC1937, HCC1143, MDA-MB-468 and HCC38) and 2 (BL2; SUM149PT, HCC70, HCC1806), mesenchymal (M; BT549) and mesenchymal-like (MSL; Hs578T, SUM159PT, MDA-MB-436, MDA-MB-231) and luminal androgen receptor (LAR; MDA-MB-453, SUM185PE) subtypes, as defined by Lehmann *et al*.^15^. Additional cell lines that were not previously classified in subtypes were also used (BT20, MDA-MB-435s, SKBR7, SUM1315M02, SUM229PE and SUM52PE). Cell lines from the immunomodulatory subtype were not included, as these cell lines (DU4775, HCC1187) grow in (mixed) suspension. Other TNBC cell lines, including CAL-51, CAL-120, CAL-148, CAL-851, HCC1395, HCC1599, HCC2157, HCC2185, HCC3153, HDQ-P1, MDA-MB-157, MFM-223 and SW527 were not used in this study due to technical considerations (e.g. limited proliferation, growing in (mixed) suspension cultures and availability). Cell line mutations were derived from the COSMIC database cell line project^31^.

### Reagents and antibodies

For screening and primary validations with MEK and Akt inhibitors, a library of 378 kinase inhibitors was used (L1200 library; Selleckchem, Huissen, The Netherlands, Supplementary Dataset 1). A concentration of 1 µM kinase inhibitor was used for the primary screening in the 20 TNBC cell lines. For further validation and other experiments, selumetinib (S1008), AZD8330 (S2134), PD184352 (S1020), MK-2206 (S1078), AZD5363 (S8019) and ipatasertib (GDC-0068; S2808) were purchased from Selleckchem.

The mouse antibody against MEK1/2 (4694S) and rabbit antibodies against phospho-MEK1/2 (Ser217/221, 9120), phospho-Akt (Ser473, 9271), phospho-p44/42 MAPK (ERK1/2, Thr202/Tyr204, 9101), phospho-mTOR (Ser2448, 5536S), Akt (9272), p44/42 MAPK (Erk1/2, 4695) were purchased from Cell Signaling (Leiden, The Netherlands). The mouse antibody against α-tubulin (T-9026) was from Sigma-Aldrich. Secondary goat antibodies horseradish peroxidase (HRP)-linked anti-rabbit (111-035-003) and anti-mouse (115-035-003) and Alexa-647-linked anti-mouse (115-605-146) were purchased from Jackson Immunoresearch (Bio-Connect, Huissen, The Netherlands).

### Cell proliferation assays

Cell proliferation was determined using a sulforhodamine B (SRB) colorimetric assay as described previously^32^. TNBC cell lines were plated at optimal cell densities ranging from 4,000 to 18,000 cells in 96-well plates and allowed to attach overnight before treatment. Cells were treated with kinase inhibitors for 4 days and thereafter fixed by adding 30 µL 50% trichloroacetic acid (TCA; Sigma-Aldrich). Cellular proteins were stained with 0.4% SRB (Sigma-Aldrich) and unbound SRB was removed with 1% acetic acid (VWR, Amsterdam, The Netherlands). Protein-bound SRB was solubilised in 10 mM aqueous unbuffered Tris (Fischer Scientific) and SRB absorbance was measured at 540 nm on an Infinite M1000 microplate reader (Tecan, Giessen, The Netherlands). Dose response curves and IC50 values were generated in GraphPad Prism (version 7.01).

### Protein extraction and western blotting

Protein extraction and western blotting were conducted as described before^33^. Cellular proteins (20 µg/lane) were loaded on 7.5% polyacrylamide gels for SDS-PAGE. After electrophoresis, proteins were transferred to polyvinylidene fluoride (PVDF) membranes (Milipore, Amsterdam, The Netherlands) overnight and blocked in 5% bovine serum albumin (BSA; Sigma Aldrich) in Tris-buffered saline with 0.05% Tween (TBS-T_0.05_). Primary and secondary antibodies were dissolved in 1% BSA in TBS-T_0.05_. Using an Amersham Imager 600 (GE Healthcare Life Sciences, Eindhoven, The Netherlands) Alexa Fluor 647-conjugated antibodies were visualised by Cy5 fluorescence and HRP-conjugated antibodies by chemiluminescence after staining with ECL (Prime) Western Blotting Detection Reagent (GE Healthcare Life Sciences). Signal intensities were quantified using ImageJ.

### Gene set enrichment analysis (GSEA) of TNBC cell lines

TNBC cell line mRNA microarray data were previously established^34^. In short, raw data (.CEL files) were processed using the robust multi-array average (RMA) method and data are available in the Gene Expression Omnibus data repository (Accession number: GSE41313). Protein abundance in the TNBC cell lines was previously analysed using high-end proteomics (Manuscript in preparation, data available upon request). Data were log-transformed (log_2_) and gene set enrichment was evaluated using GSEA software^35,36^. Enrichment of canonical pathways was tested using the curated c2cp gene sets from KEGG and Reactome (1,000 permutations per gene set). To visualise the significantly enriched gene sets (P < 0.005, FDR Q-value < 0.1) Cytoscape was used to organise these in enrichment maps (similarity cut-off >0.7)^37^. For all the enriched gene sets involved in cell cycle, the genes that were core enriched (signal-to-noise score >0.1) were collected and used for plotting heatmaps of their expression in TNBC cell lines (mRNA and protein expression) and breast cancer patients (mRNA). Overlapping networks of these genes from mRNA and protein expression were made by retrieving their network in STRING (v10.0) and providing colour annotation in Cytoscape. Pathway mapping of the core enriched genes was performed in Ingenuity Pathway Analysis (IPA) software (QIAGEN Bioinformatics).

### Gene expression in breast cancer patients

RNA sequencing and clinical data of breast cancer patients were retrieved from the TCGA database in R using the TCGAbiolinks package from Bioconductor^38^. Normalised RNA sequencing data from Illumina HiSeq platforms from primary solid tumour samples of the TCGA-BRCA project were downloaded. Cell cycle genes that were established as described in the previous section were used for generating heatmaps for both all breast cancer (n = 1093) and TNBC patients (n = 120) specifically. TNBC patients were selected on the absence of HER2, ER and PR, as annotated in the TCGA data.

### Statistical analysis

All heatmaps and hierarchical clusters were generated using the CRAN pheatmap package in R studio (version 3.3.1)^39^. Statistical analysis of grouped western blot outcomes with replicated measurements was performed using a two-way ANOVA in GraphPad Prism (Version 7). Significance was set at P < 0.05. IC50 values were also calculated in GraphPad Prism.

## Supplementary information

Supplementary Figures and Tables

Supplementary Dataset 1

Supplementary Dataset 2

## Data Availability

The cell line transcriptomics and TNBC patient gene expression datasets analysed in this study were available from the GEO data repository (GEO: GSE41313) and TCGA-BRCA database respectively. The proteomics dataset analysed in the current study is available from the corresponding author upon reasonable request. All other data are included in this article and its Supplementary Files.
