# Supplementary material for: An increased cell cycle gene network determines MEK and Akt inhibitor double resistance in triple-negative breast cancer
Source: Sci Rep. 2019 Sep 16;9:13308. doi: 10.1038/s41598-019-49809-3 (PMC6746778; doi:10.1038/s41598-019-49809-3)
Supplement: Supplementary file 1 — Supplementary Figures and Tables [file 41598_2019_49809_MOESM1_ESM.pdf]

Supplementary material for:

An increased cell cycle gene network determines MEK and Akt inhibitor double resistance in triple-negative breast cancer.

Vera E. van der Noord[1, 3], Ronan P. McLaughlin[1, 3], Marcel Smid[2], John A. Foekens[2], John W.M. Martens[2], Yinghui Zhang[1], Bob van de Water[1, *]

[1]Division of Drug Discovery and Safety, Leiden Academic Center for Drug Research, Leiden University, PO box 9502, 2300 RA Leiden, The Netherlands

[2]Department of Medical Oncology, Erasmus MC Cancer Institute, Erasmus University Medical Center, PO Box 2040, 3000 CA Rotterdam, The Netherlands

[3]These authors contributed equally to the work.

*Corresponding author: Professor Bob van de Water, Division of Drug Discovery and Safety, Leiden Academic Center for Drug Research, Leiden University, Einsteinweg 55, PO box 9502, 2300 RA Leiden, The Netherlands. Email: b.water@lacdr.leidenuniv.nl

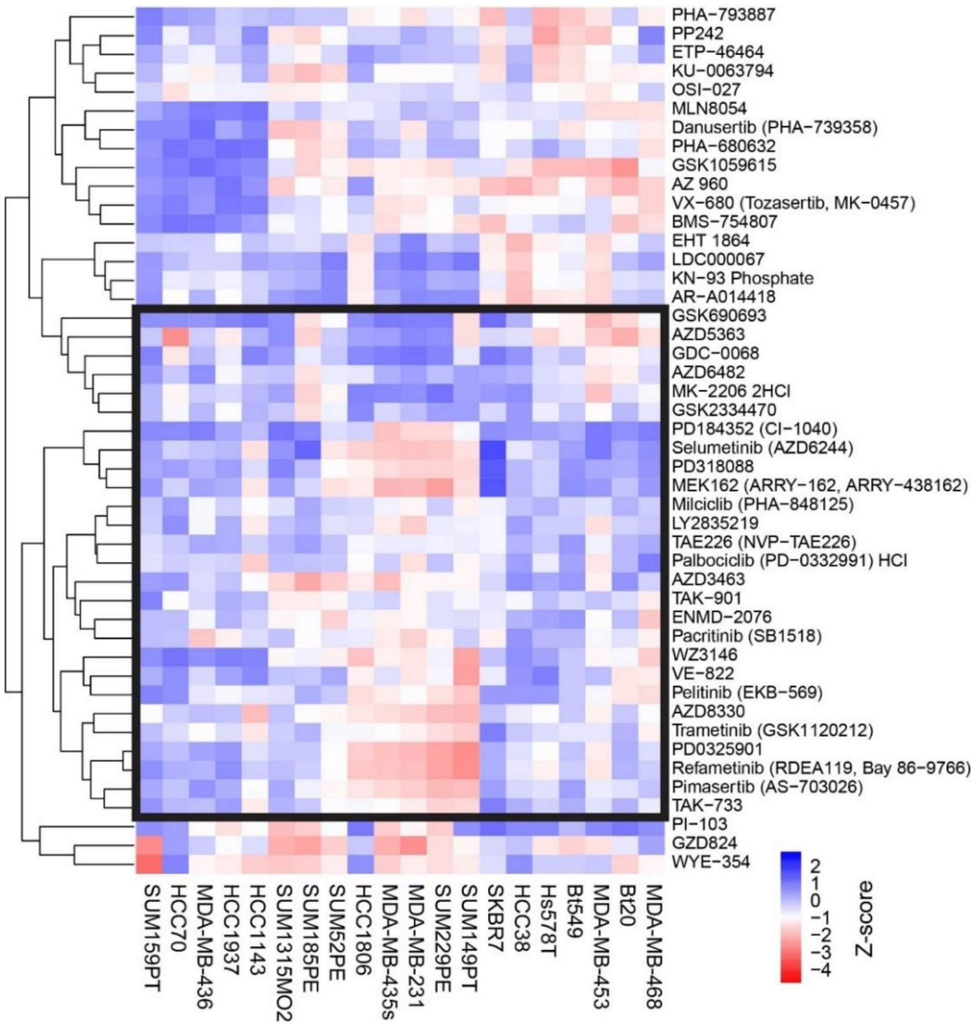

Supplementary Figure S1. Clustered kinase inhibitors differentially affecting TNBC cell proliferation. Enlarged and annotation version of the clustered branch of differentially effective kinase inhibitors from Figure 1A. The black box highlights mainly MEK and Akt inhibitors. Low Z-scores (red) indicate reduced cell proliferation, whereas high Z-scores indicate resistance (blue) upon treatment with the kinase inhibitors (1 µM).

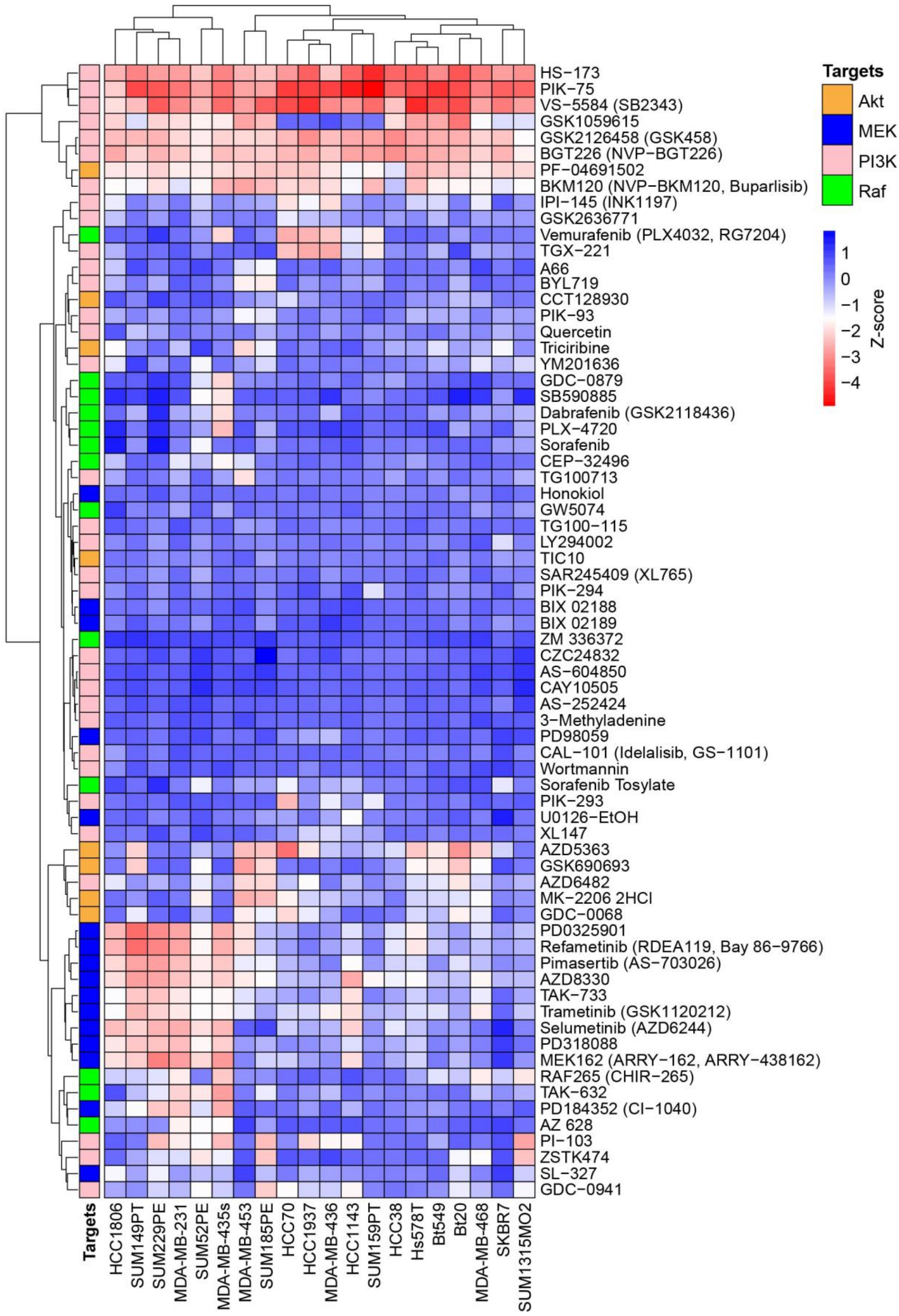

Supplementary Figure S2. Proliferative responses to MEK, Akt, Raf and PI3K inhibitors (1 μM) among TNBC cell lines. Low Z-scores (red) indicate reduced cell proliferation, whereas high Z-scores indicate resistance (blue) upon treatment with the kinase inhibitors.

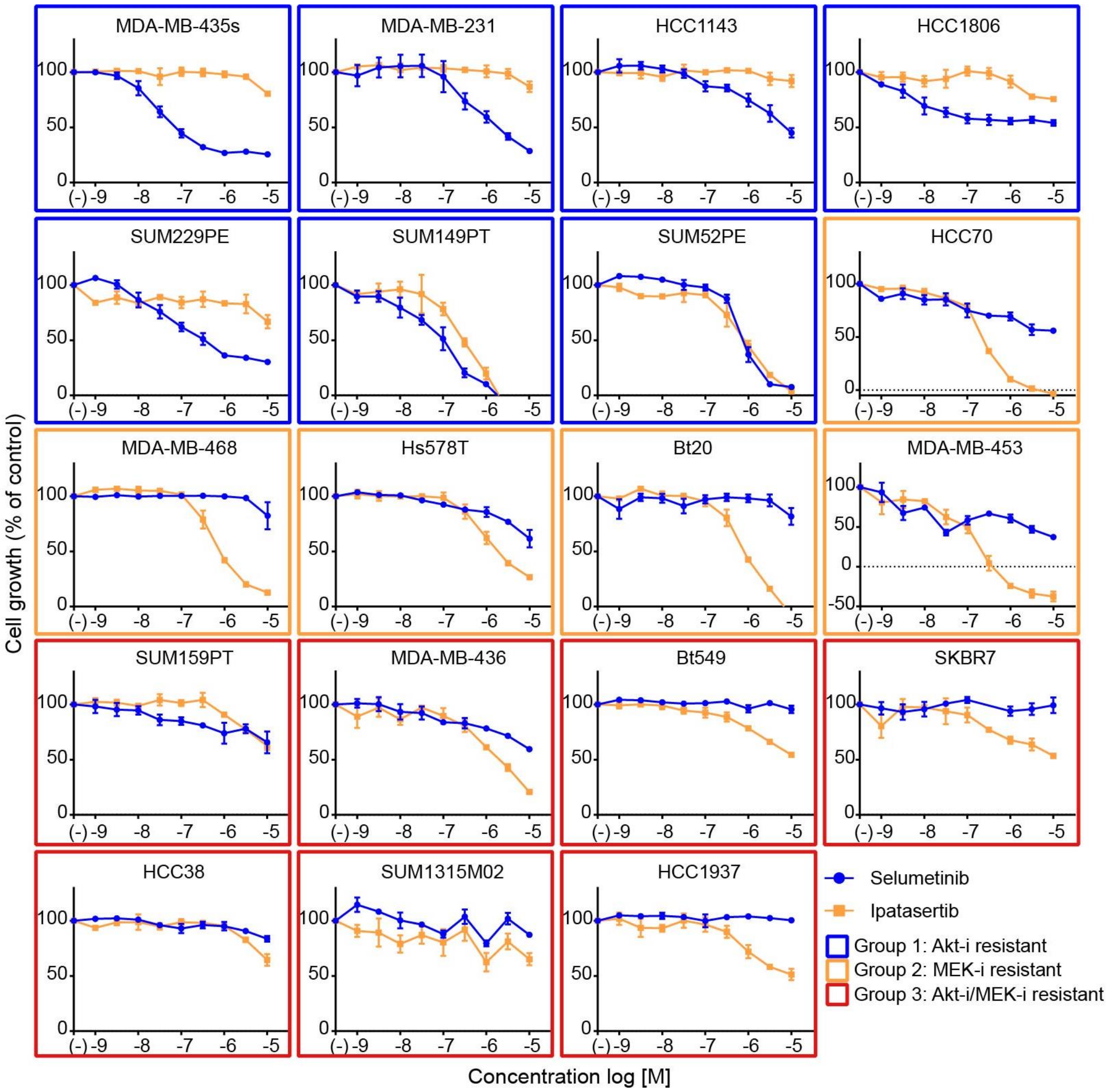

Supplementary Figure S3. Sensitivity profiling in TNBC cell lines. Validation of Akt-i/MEK-i sensitivity classifications from primary screening (Figure 1B). Proliferative responses of cell lines to increasing concentrations (0.001-10 μM) of selumetinib (MEK-i) and ipatasertib (Akt-i) are shown.

Source images Figure 2

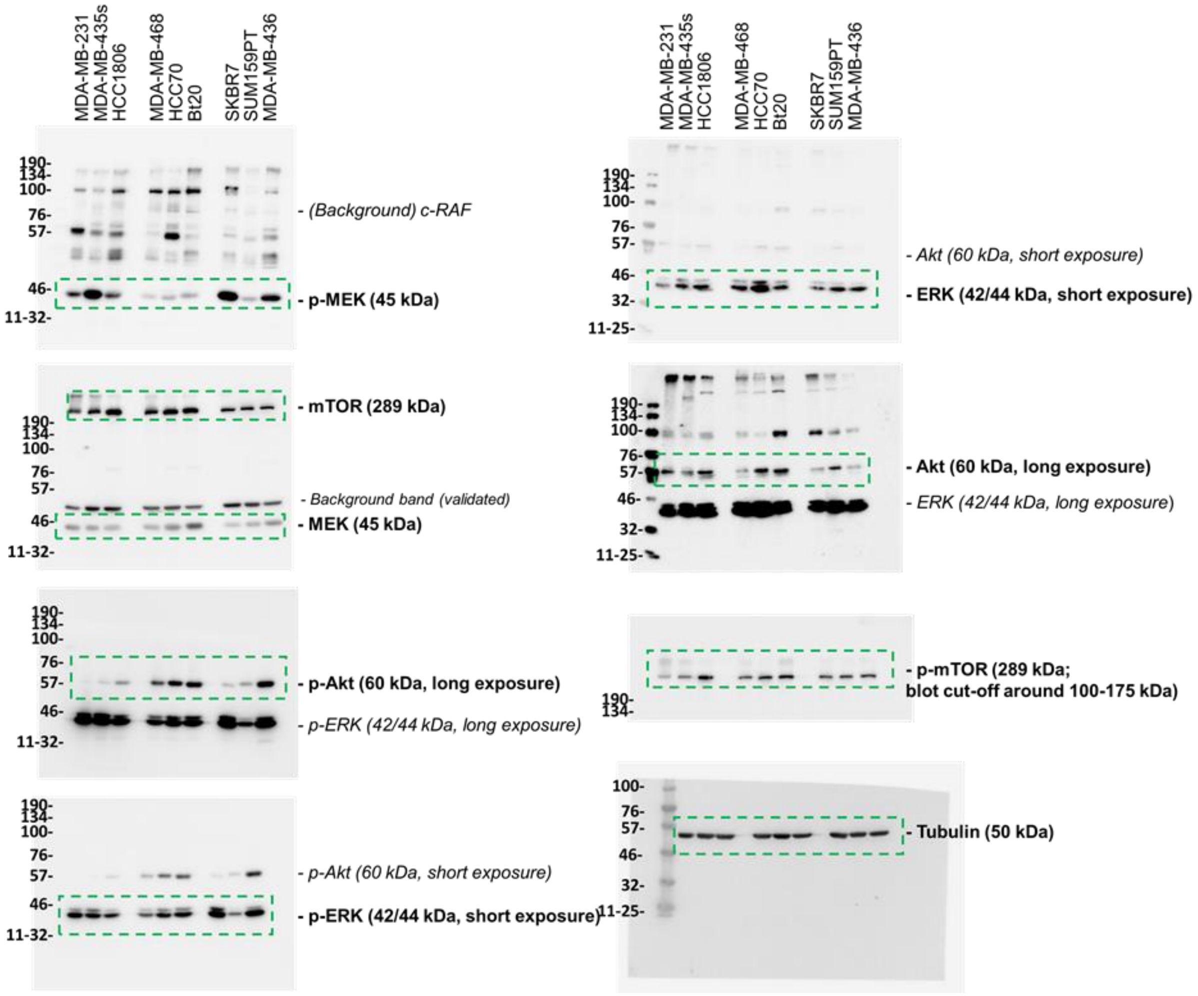

Supplementary Figure S4. Uncropped full-length images of blots. Source images for the western blot bands presented in Figure 2, 3 and 6. Green dotted squares indicate the approximate cropping area. Antibody combinations were previously tested to not interfere with each others bands. In some cases, blots were cut horizontally to perform separate stainings and full-length blots are not depicted. Protein sizes are according to pre-stained protein ladders from Cell signalling (#13953) or Thermo Fisher (#26619).

Source images Figure 3

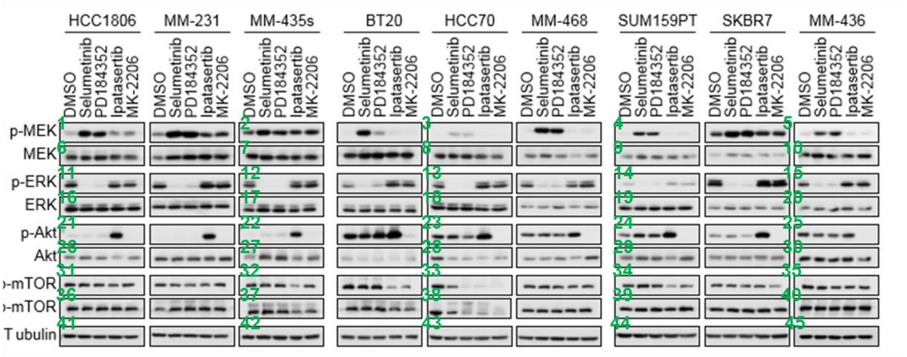

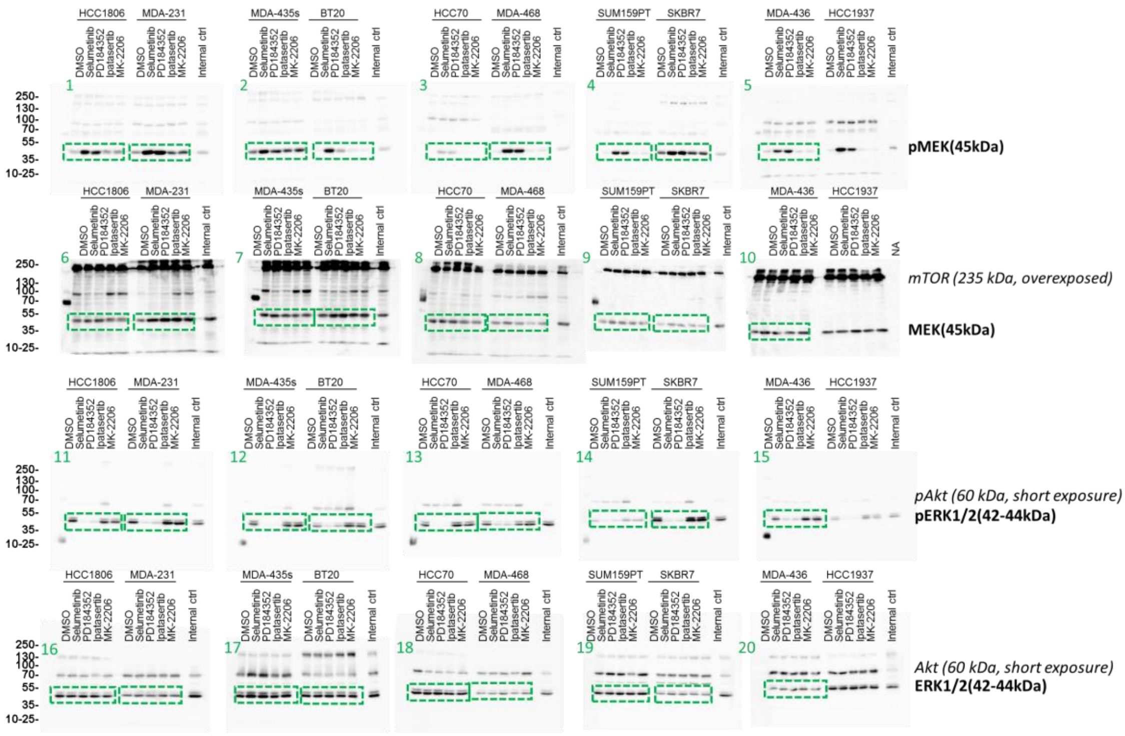

Supplementary Figure S4. Uncropped full-length images of blots (continued). Source images for the western blot bands presented in Figure 2, 3 and 6. Green dotted squares indicate the approximate cropping area. Antibody combinations were previously tested to not interfere with each others bands. In some cases, blots were cut horizontally to perform separate stainings and full-length blots are not depicted. Protein sizes are according to pre-stained protein ladders from Cell signalling (#13953) or Thermo Fisher (#26619).

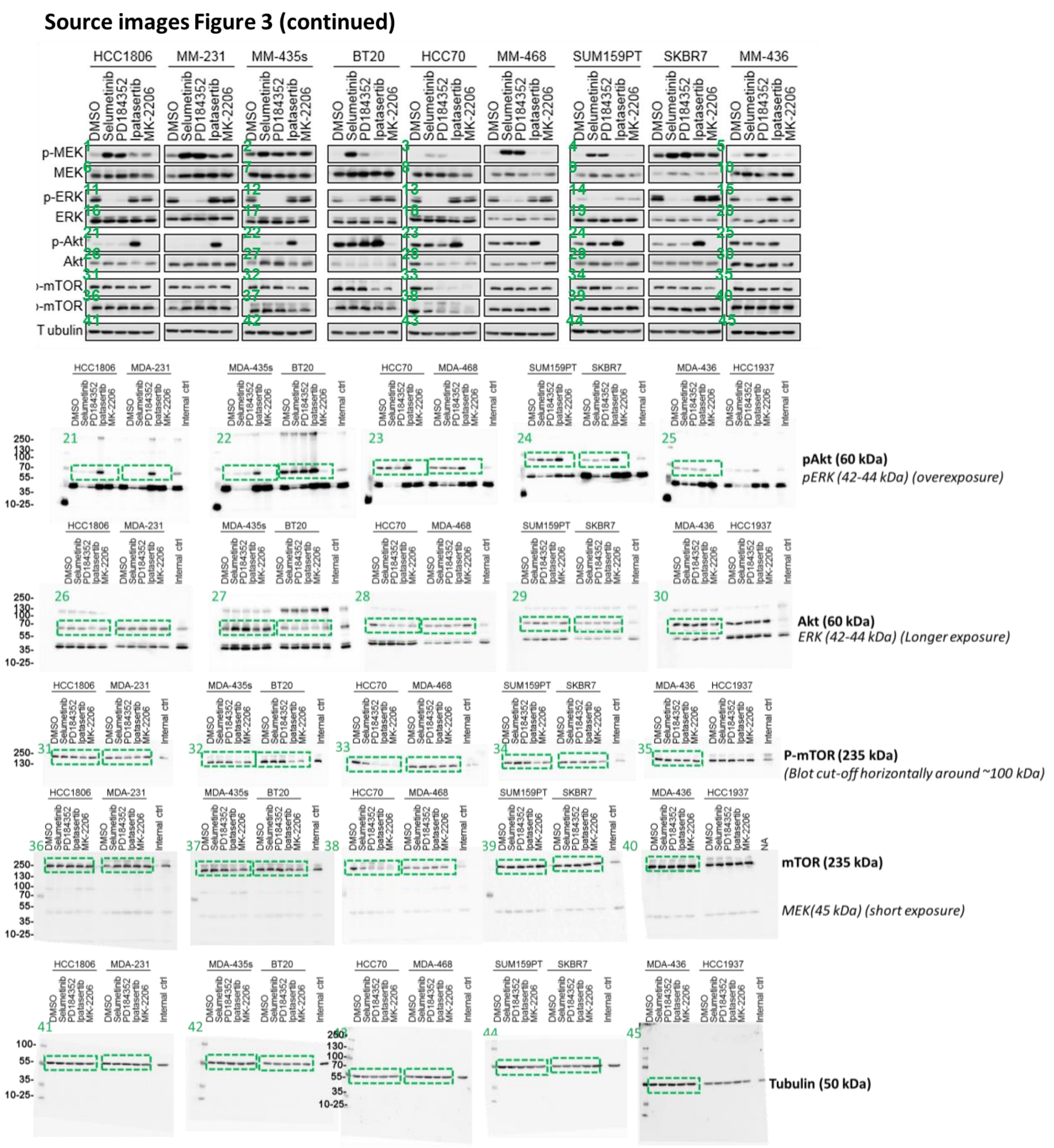

Supplementary Figure S4. Uncropped full-length images of blots (continued). Source images for the western blot bands presented in Figure 2, 3 and 6. Green dotted squares indicate the approximate cropping area. Antibody combinations were previously tested to not interfere with each others bands. In some cases, blots were cut horizontally to perform separate stainings and full-length blots are not depicted. Protein sizes are according to pre-stained protein ladders from Cell signalling (#13953) or Thermo Fisher (#26619).

Source images Figure 6:

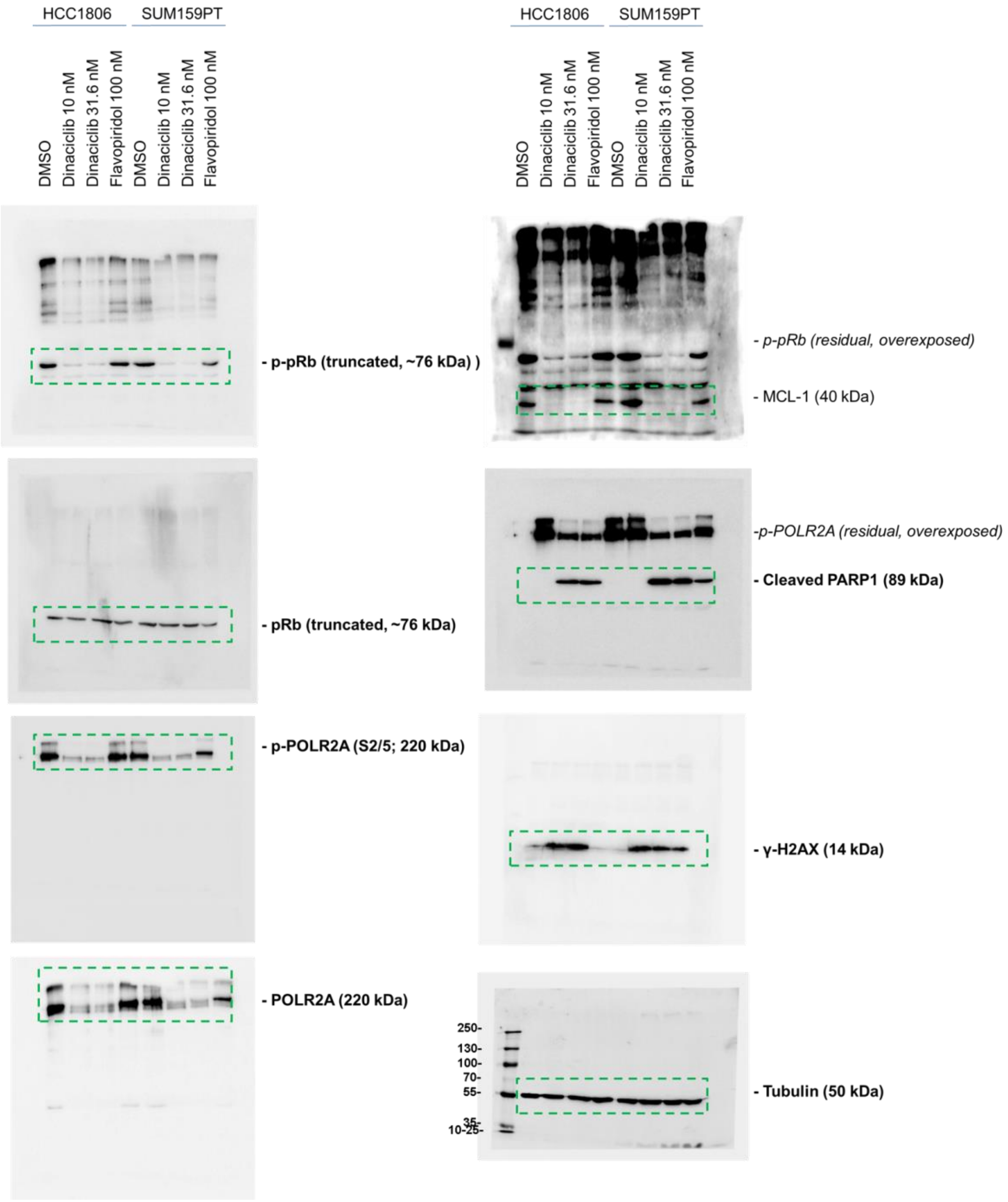

Supplementary Figure S4. Uncropped full-length images of blots (continued). Source images for the western blot bands presented in Figure 2, 3 and 6. Green dotted squares indicate the approximate cropping area. Antibody combinations were previously tested to not interfere with each others bands. In some cases, blots were cut horizontally to perform separate stainings and full-length blots are not depicted. Protein sizes are according to pre-stained protein ladders from Cell signalling (#13953) or Thermo Fisher (#26619).

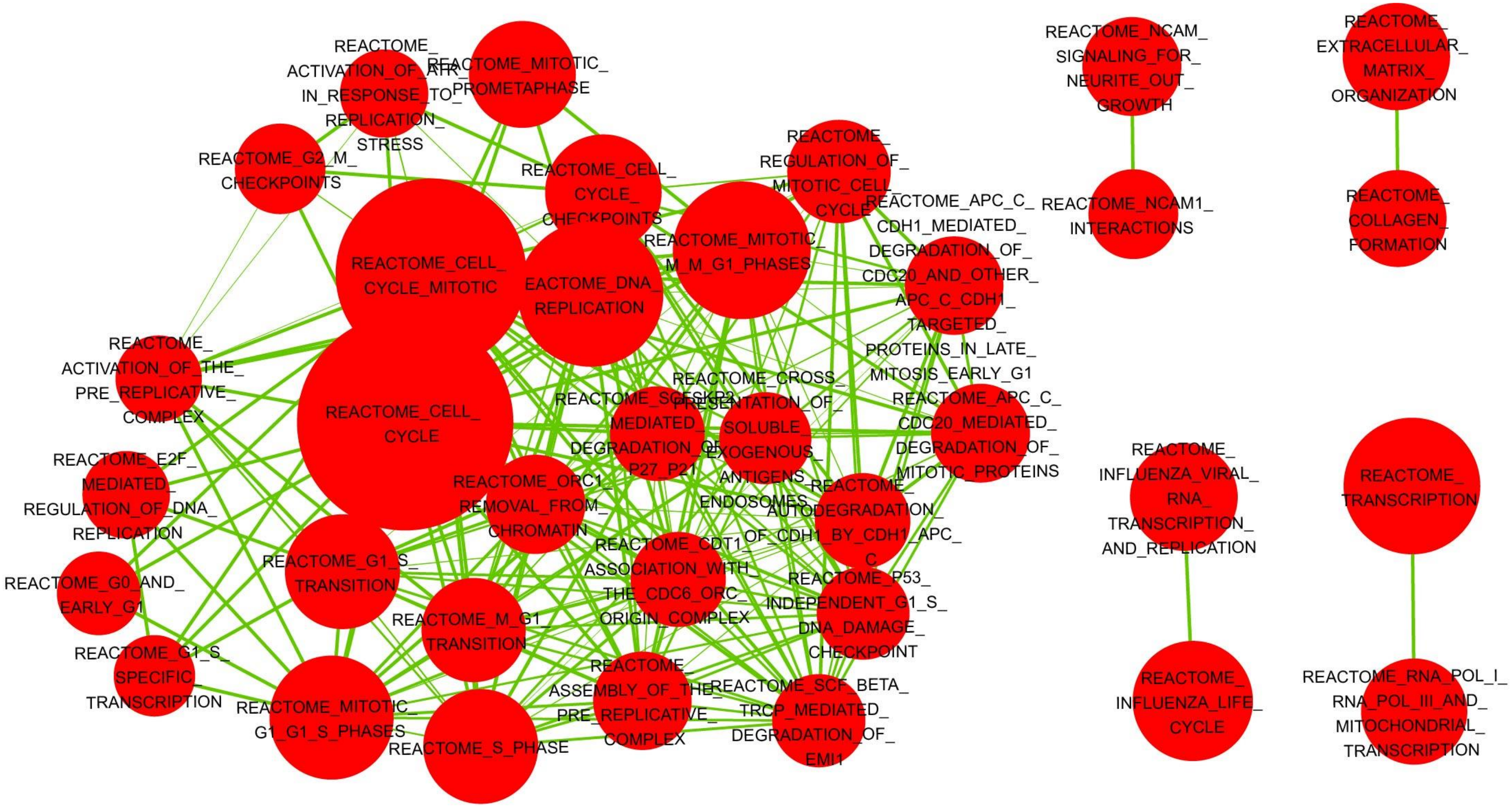

Supplementary Figure S5. Gene set enrichment map for sets enriched in mRNA expression for Akt-i/MEK-i-resistant Group 3 cell lines. This figure represents an enlarged and annotated version of Figure 4A (left part). mRNA expression was compared between MEK and Akt inhibitor-resistant Group 3 versus MEK or Akt inhibitor-resistant Group 1 and 2 cell lines using gene set enrichment analysis (GSEA). The enrichment map visualises enriched gene sets (P<0.005, FDR Q-value<0.1, overlap>0.7), among which cell cycle gene sets were most abundant. Every node (red) represents a single overexpressed gene set and the size of the node indicates the size of the gene set. Every line (green) represents overlap between gene sets.

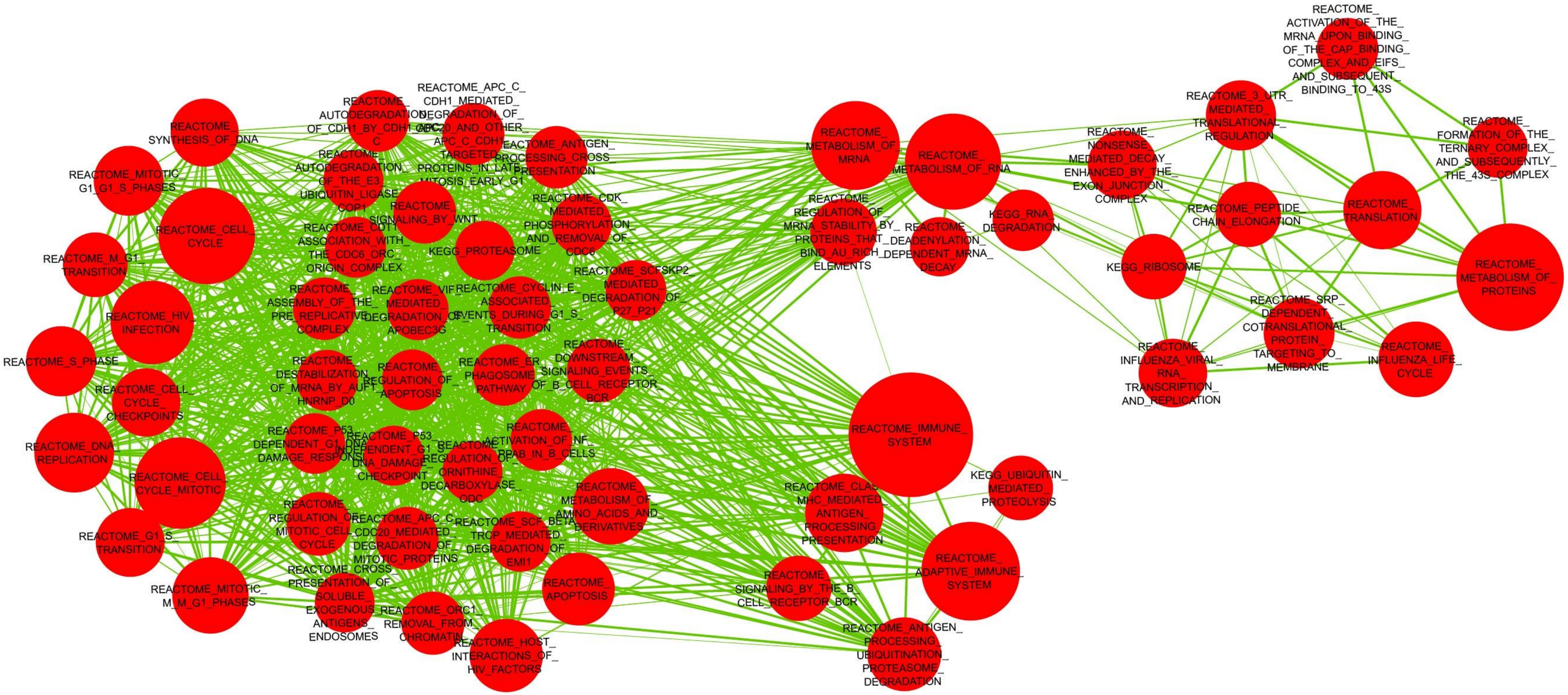

Supplementary Figure S6. Gene set enrichment map for sets enriched in protein expression for Akt-i/MEK-i-resistant Group 3 cell lines. This figure represents an enlarged and annotated version of Figure 4A (right part). Protein expression was compared between MEK and Akt inhibitor-resistant Group 3 versus MEK or Akt inhibitor-resistant Group 1 and 2 cell lines using gene set enrichment analysis (GSEA). The enrichment map visualises enriched gene sets (P<0.005, FDR Q-value<0.1, overlap>0.7), among which cell cycle gene sets were most abundant. Every node (red) represents a single overexpressed gene set and the size of the node indicates the size of the gene set. Every line (green) represents overlap between gene sets.

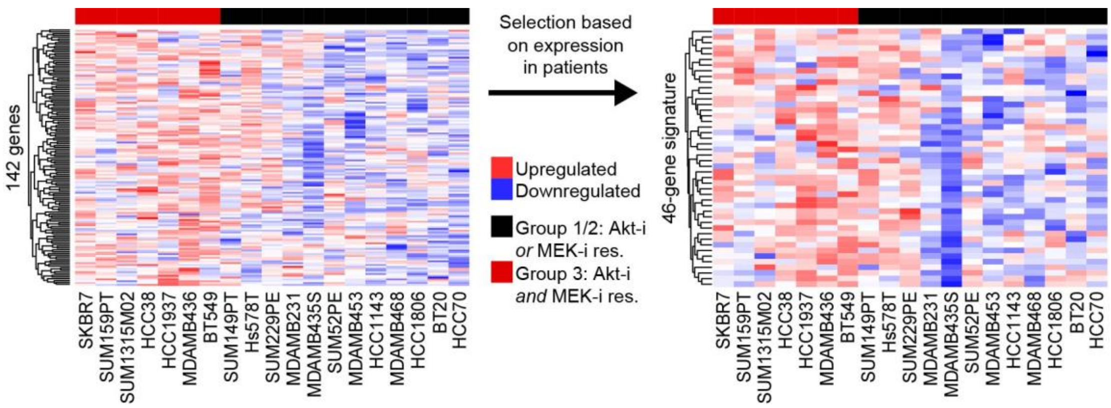

Supplementary Figure S7. Expression of the 142 cell cycle genes and the selected 46 clinically differentially expressed genes in TNBC cell lines. The mRNA expression of the 142 cell cycle genes, as given in Figure 4B, and of the later selected 46 clinically differentially expressed genes, as shown in Figure 5, in the TNBC cell lines is shown.

Supplementary Table S1. IC50's for Akt and MEK inhibitors. To validate the resistance to Akt and/or MEK inhibitors, cells were treated with a dose range (0.01 – 3.16 µM) of inhibitors in three technical replicates in two independent experiments. Proliferation was evaluated using an SRB assay and IC50's were calculated using GraphPad Prism. If 50% of proliferation was not reached, IC50's are noted as >3.16. For the case of more than 50% reduction of growth at the lowest concentration, IC50's were noted as <0.01.

		**Akt-i resistant**			**MEK-i resistant**			**Akt-i & MEK-i double resistant**		
	IC50 (µM)	**MDA-MB-231**	**MDA-MB-435s**	**HCC1806**	**MDA-MB-468**	**BT20**	**HCC70**	**SKBR7**	**SUM159PT**	**MDA-MB-436**
**Akt inhibitors**	**MK-2206**	>3.16	>3.16	>3.16	0.30	>3.16	0.26	>3.16	1.35	>3.16
	**GDC-0068**	>3.16	>3.16	>3.16	0.16	0.38	0.21	>3.16	>3.16	>3.16
	**AZD5363**	>3.16	>3.16	>3.16	0.15	0.34	0.17	>3.16	~3.16	>3.16
	**GSK690693**	>3.16	>3.16	>3.16	>3.16	0.15	1.31	>3.16	>3.16	>3.16
**MEK inhibitors**	**Selumetinib**	0.05	0.03	>3.16	>3.16	>3.16	>3.16	>3.16	2.74	>3.16
	**MEK-i162**	<0.01	<0.01	7.617	>3.16	>3.16	>3.16	>3.16	~3.16	>3.16
	**Trametinib**	<0.01	<0.01	<0.01	0.03	>3.16	>3.16	>3.16	NA	NA
	**PD318088**	0.15	<0.01	<0.01	0.32	>3.16	>3.16	>3.16	>3.16	>3.16
	**PD184352**	0.79	0.12	<0.01	>3.16	>3.16	>3.16	>3.16	>3.16	>3.16
	**PD032590**	<0.01	<0.01	0.48	0.53	>3.16	>3.16	>3.16	>3.16	>3.16
	**Pimasertib**	<0.01	<0.01	2.27	2.39	>3.16	>3.16	>3.16	>3.16	>3.16
	**Refametinib**	<0.01	<0.01	<0.01	>3.16	>3.16	>3.16	>3.16	~3.16	>3.16
	**TAK-733**	<0.01	<0.01	<0.01	0.27	>3.16	>3.16	>3.16	~3.16	>3.16
	**AZD8330**	<0.01	<0.01	<0.01	>3.16	>3.16	>3.16	>3.16	>3.16	>3.16

Supplementary Table S2. Common mutations in cell lines in components of the PI3K, MAPK, cell cycle and DNA damage repair pathways. Mutations in Cancer Gene Census genes were deducted from the COSMIC database (http://cancer.sanger.ac.uk/cosmic).

		PI3K	MAPK	PI3K/ MAPK	Cell cycle	DNA damage repair
1): Akt-i resistant	**HCC1143**			FGFR2	TP53	
	**MDA-MB-435s**		BRAF		TP53, CDKN2A, EP300	
	**MDA-MB-231**		BRAF,NF1	PDGFRA, KRAS	TP53, CDKN2A	
	**HCC1806**		NFATC2, ROS1	PDGFRB	TP53, CDKN2A	
	**SUM229PE**			KRAS	CDKN2A	
	**SUM52PE**				TP53, CDKN2A	
	**SUM149PT**				TP53, CDKN2A, EP300	BRCA1
2) MEK-i resistant	**HCC70**	PTEN			TP53, RB1	
	**MDA-MB-453**	PIK3CA, PTEN	ROS1		CREBBP, ATM, STAG2	CREBBP, ATM
	**SUM185PE**	PIK3CA			TP53	
	**BT20**	PIK3CA			ATM, CDKN2C, CDKN2A, RB1, TP53	MLH1
	**Hs578T**	PIK3R1	NF1	HRAS	TP53, CDKN2A, POLE	POLE
	**MDA-MB-468**	PTEN	CACNA1D, MAP3K13		TP53, CDKN2A, RB1	
3) Akt/MEK-i resistant	**BT549**	PTEN		FGFR1	TP53, RB1, BUB1B, (DCTN1), PCM1	MUTYH
	**SKBR7**			KRAS, NRAS	CDKN2A	
	**HCC1937**	PTEN	MAPK13		TP53, RB1	BRCA1
	**MDA-MB-436**	PIK3R1			TP53, RB1	BRCA1, POLE
	**HCC38**		IKBKB		TP53, CDKN2A	PMS2, BRCA1*
	**SUM159PT**	PIK3CA		HRAS	TP53	
	**SUM1315MO2**	PIK3CA			TP53	BRCA1

* methylation

Supplementary Table S3. Enriched cell cycle genes in Akt-i/MEK-i resistant group 3 cell lines. From the significantly enriched, cell cycle associated, gene sets, enriched genes or proteins (signal-to-noise < 0.1) were used for further analysis.

mRNA expression		Protein expression		Shared		
PROBE	Signal-to-noise	PROBE	Signal-to-noise	PROBE	Signal-to-noise (mRNA)	Signal-to-noise (protein)
1. **SNAP25**	0.710	1. **SEC13**	0.797	1. **PSMD11**	0.447	0.631
2. **STX11**	0.640	2. **RUVBL1**	0.765	2. **PSMC2**	0.379	0.632
3. **KAT2B**	0.530	3. **PSMC4**	0.737	3. **SEC13**	0.124	0.797
4. **PPP2R3B**	0.528	4. **PSMD3**	0.737	4. **PSMD3**	0.107	0.737
5. **SGOL1**	0.499	5. **PSMA3**	0.693	5. **PSMD1**	0.106	0.678
6. **PTRF**	0.489	6. **PSMD1**	0.678	6. **PCM1**	0.409	0.336
7. **TUBB4A**	0.448	7. **PSMA4**	0.670	7. **PSMD12**	0.103	0.611
8. **PSMD11**	0.447	8. **CKAP5**	0.666	8. **PSMA7**	0.139	0.574
9. **CDC25A**	0.425	9. **YWHAG**	0.647	9. **PSMB7**	0.117	0.571
10. **STX2**	0.423	10. **PSMC2**	0.632	10. **ORC5**	0.240	0.424
11. **PCM1**	0.409	11. **PSMD11**	0.631	11. **RPS27**	0.108	0.545
12. **CENPI**	0.393	12. **PSMD12**	0.611	12. **PSMD9**	0.375	0.239
13. **PSMC2**	0.379	13. **RUVBL2**	0.604	13. **CCNB1**	0.170	0.415
14. **PSMD9**	0.375	14. **PSMD4**	0.589	14. **CDC23**	0.267	0.308
15. **NINL**	0.360	15. **PSMA7**	0.574	15. **GINS4**	0.143	0.414
16. **POLR3D**	0.332	16. **PSMB7**	0.571	16. **ACTR1A**	0.150	0.375
17. **RFC3**	0.316	17. **PSMA1**	0.569	17. **CUL1**	0.164	0.338
18. **HIST1H2AC**	0.308	18. **SKP1**	0.557	18. **ANAPC7**	0.141	0.338
19. **CENPO**	0.298	19. **RPS27**	0.545	19. **NDC80**	0.104	0.360
20. **PPP2R1B**	0.293	20. **CDK4**	0.533	20. **CDK2**	0.138	0.316
21. **RPA4**	0.289	21. **PSME2**	0.526	21. **CDK5RAP2**	0.180	0.255
22. **GTF3C3**	0.284	22. **PSMC5**	0.514	22. **TYMS**	0.228	0.198
23. **GTF2H2**	0.283	23. **PSMD8**	0.504	23. **SPC25**	0.209	0.147
24. **SNAPC1**	0.277	24. **RBX1**	0.498	24. **PPP2CB**	0.107	0.223
25. **TUBA1A**	0.274	25. **NUDC**	0.487	25. **ZWILCH**	0.154	0.176
26. **POLR1A**	0.274	26. **PSMC1**	0.478	26. **ERCC6L**	0.175	0.127
27. **CDK6**	0.269	27. **PSME1**	0.478	27. **KIF2A**	0.117	0.143
28. **CDC23**	0.267	28. **PSMB4**	0.472	28. **GINS1**	0.115	0.141
29. **LIN54**	0.250	29. **PSMD13**	0.466	29. **SYNE1**	0.112	0.120
30. **STX4**	0.245	30. **DCTN2**	0.445	30. **MAX**	0.116	0.116
31. **CEP250**	0.244	31. **RPS27A**	0.443			
32. **ORC5**	0.240	32. **CLASP1**	0.442			
33. **CDC6**	0.235	33. **UBE2E1**	0.440			
34. **TFDP1**	0.232	34. **MAGED1**	0.438			
35. **MCM6**	0.232	35. **DCTN3**	0.435			
36. **ATRIP**	0.229	36. **ORC5**	0.424			
37. **TYMS**	0.228	37. **UBE2I**	0.423			
38. **WRAP53**	0.223	38. **CUL5**	0.422			
39. **NUP43**	0.222	39. **CCNB1**	0.415			
40. **BUB1**	0.220	40. **GINS4**	0.414			

**41.**	**BRCA1**	0.218	**41.**	**PSMD7**	0.382
**42.**	**VAMP1**	0.218	**42.**	**ACTR1A**	0.375
**43.**	**E2F1**	0.211	**43.**	**PAFAH1B1**	0.365
**44.**	**STX1B**	0.210	**44.**	**PSMB1**	0.364
**45.**	**SPC25**	0.209	**45.**	**PSMC3**	0.361
**46.**	**TUBG1**	0.205	**46.**	**NDC80**	0.360
**47.**	**ERCC6**	0.205	**47.**	**PSMD6**	0.355
**48.**	**SKA1**	0.201	**48.**	**CLIP1**	0.352
**49.**	**CCNE1**	0.199	**49.**	**XPO1**	0.347
**50.**	**ORC1**	0.199	**50.**	**ANAPC2**	0.338
**51.**	**TAOK1**	0.192	**51.**	**CUL1**	0.338
**52.**	**CCNE2**	0.190	**52.**	**PCM1**	0.336
**53.**	**HIST1H4J**	0.187	**53.**	**ANAPC7**	0.333
**54.**	**GTF2H2B**	0.187	**54.**	**LMNA**	0.331
**55.**	**CCNA2**	0.184	**55.**	**KIF2C**	0.328
**56.**	**CDKN2C**	0.181	**56.**	**CDK2**	0.316
**57.**	**CDK5RAP2**	0.180	**57.**	**TUBGCP2**	0.314
**58.**	**TUBGCP5**	0.179	**58.**	**TCEB2**	0.309
**59.**	**BTRC**	0.178	**59.**	**CDC23**	0.308
**60.**	**PSMB2**	0.177	**60.**	**PSMB5**	0.288
**61.**	**ERCC6L**	0.175	**61.**	**DYNLL1**	0.279
**62.**	**CENPK**	0.172	**62.**	**PSMB8**	0.277
**63.**	**CCNB1**	0.170	**63.**	**CDK5RAP2**	0.255
**64.**	**POLE2**	0.169	**64.**	**PSMD2**	0.251
**65.**	**CEP135**	0.167	**65.**	**NUF2**	0.248
**66.**	**CUL1**	0.164	**66.**	**PSMD9**	0.239
**67.**	**RBBP4**	0.163	**67.**	**CETN2**	0.233
**68.**	**DNA2**	0.161	**68.**	**PSMC6**	0.232
**69.**	**DIDO1**	0.160	**69.**	**DYNC1I2**	0.232
**70.**	**PPP2R2A**	0.156	**70.**	**KIF20A**	0.226
**71.**	**CEP72**	0.156	**71.**	**RPA1**	0.226
**72.**	**ZWILCH**	0.154	**72.**	**PPP2CB**	0.223
**73.**	**HAUS2**	0.152	**73.**	**HSPA2**	0.222
**74.**	**CENPH**	0.151	**74.**	**PSMD10**	0.217
**75.**	**CDC25C**	0.150	**75.**	**PPP2R1A**	0.206
**76.**	**ACTR1A**	0.150	**76.**	**FEN1**	0.200
**77.**	**TAF1B**	0.147	**77.**	**TYMS**	0.198
**78.**	**MCM10**	0.147	**78.**	**MNAT1**	0.178
**79.**	**UBTF**	0.145	**79.**	**PPP2R5D**	0.177
**80.**	**MCM4**	0.144	**80.**	**ZWILCH**	0.176
**81.**	**CDC27**	0.144	**81.**	**ANAPC5**	0.176
**82.**	**ANAPC10**	0.143	**82.**	**PRKACA**	0.166
**83.**	**LIN52**	0.143	**83.**	**ORC2**	0.166
**84.**	**GINS4**	0.143	**84.**	**SEH1L**	0.155
**85.**	**SNAPC3**	0.142	**85.**	**YWHAE**	0.153
**86.**	**SNAPC4**	0.142	**86.**	**DCTN1**	0.150
**87.**	**SGOL2**	0.142	**87.**	**PSMA2**	0.150
**88.**	**PLK4**	0.141	**88.**	**HSP90AA1**	0.149

**89. ANAPC7**	0.141
**90. MIS18A**	0.139
**91. PSMA7**	0.139
**92. CDK2**	0.138
**93. ZW10**	0.138
**94. KIF18A**	0.138
**95. CDCA8**	0.136
**96. CCDC99**	0.135
**97. PRIM2**	0.134
**98. CEP41**	0.134
**99. TFAM**	0.134
**100.CDC20**	0.132
**101.POLR3H**	0.129
**102.CEP76**	0.126
**103.POLA1**	0.126
**104.AURKA**	0.125
**105.BIRC5**	0.124
**106.SEC13**	0.124
**107.CEP63**	0.123
**108.SMC1A**	0.123
**109.POLR1D**	0.122
**110.BRF2**	0.119
**111.KIF2A**	0.117
**112.PSMB7**	0.117
**113.B9D2**	0.117
**114.SPC24**	0.117
**115.MAX**	0.116
**116.STX1A**	0.116
**117.CEP164**	0.115
**118.SMARCA5**	0.115
**119.CDKN2A**	0.115
**120.PLK1**	0.115
**121.ORC3**	0.115
**122.GINS1**	0.115
**123.CDC26**	0.113
**124.SYNE1**	0.112
**125.RAD17**	0.112
**126.DSN1**	0.111
**127.HIST1H2A**	0.110
**128.POLR3GL**	0.110
**129.CDK7**	0.109
**130.RPS27**	0.108
**131.PSMD3**	0.107
**132.PPP2CB**	0.107
**133.CASC5**	0.107
**134.PSMD1**	0.106
**135.MYBL2**	0.106
**136.ORC6**	0.105

**89. NUP37**	0.148
**90. SPC25**	0.147
**91. KIF2A**	0.143
**92. TERF2**	0.141
**93. GINS1**	0.141
**94. PAK2**	0.132
**95. ERCC6L**	0.127
**96. SYNE1**	0.120
**97. MAX**	0.116
**98. PSMD5**	0.112
**99. CDC16**	0.106
**100.PSMA5**	0.104
**101.DYNC1H1**	0.103
**102.MAPRE1**	0.101

**137.NDC80**	0.104
**138.SSNA1**	0.104
**139.PSMD12**	0.103
**140.SSB**	0.101
**141.MCM2**	0.100
**142.MCM8**	0.100

Supplementary Table S4. Differential sensitivity of group 3 cell lines to CDK inhibitors dinaciclib and flavopiridol. IC50 values for selected cell lines from Group 1 (Akt-i-resistant), Group 2 (MEK-i-resistant) and Group 3 (Akt-i/MEK-i double-resistant) for pan-CDK inhibitors dinaciclib and flavopiridol. IC50 values were calculated using non-linear regression fitting in GraphPad Prism.

	**Group 1 (Akt-i-resistant)**		**Group 2 (MEK-i-resistant)**		**Group 3 (Akt-i/MEK-i-resistant)**		
**IC50 (μM)**	HCC1806	MDA-MB-435s	BT20	HCC70	SKBR7	MDA-MB-436	SUM159PT
**Dinaciclib**	0.2973	0.3121	0.2069	0.1371	0.1465	0.1095	0.1543
**Flavopiridol**	0.4887	0.2758	0.149	0.1246	0.09521	0.1775	0.1768
